# Prevalence and associations between food insecurity and overweight/obesity among native Hawaiian and Pacific Islander adolescents

**DOI:** 10.1017/S1368980023000769

**Published:** 2023-07

**Authors:** Christopher R Long, Marie-Rachelle Narcisse, James P Selig, Don E Willis, Matthew Gannon, Brett Rowland, Emily S English, Pearl A McElfish

**Affiliations:** 1 University of Arkansas for Medical Sciences Northwest, College of Medicine, Fayetteville, AR 72703, USA; 2 University of Arkansas for Medical Sciences Northwest, Fay W. Boozman College of Public Health, Fayetteville, AR, USA; 3 University of Arkansas for Medical Sciences Northwest, Office of Community Health and Research, Fayetteville, AR, USA

**Keywords:** Food insecurity, Native Hawaiian and Pacific Islanders, Obesity, Adolescents

## Abstract

**Objective:**

This study estimates the prevalence of, and associations between, family food insecurity and overweight/obesity among Native Hawaiian and Pacific Islander (NHPI) adolescents and explores socio-demographic factors which might have a moderation effect on the association.

**Design:**

Cross-sectional study using 2014 NHPI-National Health Interview Survey data reported by a parent or guardian. Family-level food security was assessed by the US Department of Agriculture 10-item questionnaire. BMI for age and sex ≥ 85th and 95th percentiles defined overweight and obesity, respectively, according to US Centers for Disease Control and Prevention criteria.

**Setting:**

The USA, including all 50 states and the District of Columbia.

**Participants:**

383 NHPI adolescents aged 12–17 in the USA.

**Results:**

A third (33·5 %) of NHPI adolescents aged 12–17 were overweight (19·1 %) or obese (14·4 %); 8·1 % had low food security; and 8·5 % had very low food security. Mean family food security score was 1·06, which corresponds to marginal food security. We found no association between family food insecurity and adolescent overweight/obesity or between any other covariates and overweight/obesity, except for family Supplemental Nutrition Assistance Program (SNAP) participation. Odds of being overweight/obese were 77 % lower for adolescents in families participating in SNAP (OR: 0·23, 95 % CI: 0·08, 0·64, *P* = 0·007). The association between SNAP participation and lower odds of overweight/obesity was particularly pronounced for adolescent girls in food-insecure families.

**Conclusions:**

The association between SNAP participation and lower odds of overweight/obesity suggests potential benefit of research to determine whether interventions to increase SNAP enrollment would improve NHPI adolescents’ health outcomes.

Native Hawaiian and Pacific Islander (NHPI) families in the USA experience a high prevalence of household food insecurity relative to the overall USA^(1)^. The most recent US estimates for NHPI family food insecurity were from 2014, when 20·5 % of NHPI families were food insecure, compared to 10·6 % of all US families^(1)^. This disparity is particularly important, because NHPI adults also experience disparities related to nutrition-related chronic disease, including elevated prevalence of type 2 diabetes and obesity^(2–6)^.

Minority groups including Hispanic and African American populations have documented high prevalence of household food insecurity^(7)^ and obesity among adolescents^(8–12)^. However, prior studies have not estimated prevalence of food insecurity and obesity among NHPI adolescents using representative national data. Limited research with NHPI adolescents has suggested they experience a high prevalence of food insecurity relative to other racial and ethnic groups, although that study was conducted with adolescents from Marshallese migrant families who at the time were mostly ineligible for Supplemental Nutrition Assistance Program (SNAP) benefits^(13)^.

Studies of associations between food insecurity and adolescent overweight/obesity have yielded inconsistent findings. Although some studies have demonstrated associations between food insecurity and overweight/obesity among adolescents^(14)^, the association is often NS after adjusting for confounding factors^(15)^, and reviews incorporating multiple studies have shown differing results. Not all studies demonstrated the association between food insecurity and overweight/obesity^(16–18)^.

Factors like being a girl *v*. a boy, family SNAP participation and race and ethnicity may influence the relationship between food insecurity and adolescent overweight/obesity. Among adult women, existing research consistently demonstrates associations between food insecurity and overweight/obesity; however, results are mixed for adolescents and adult men^(16,17)^. Studies have shown household participation in SNAP, a US government programme to mitigate food insecurity for lower-income families to purchase food, can reduce the probability of overweight/obesity for children and adolescents^(19,20)^. Other studies have found evidence for an association between food insecurity and adolescent overweight/obesity among specific racial and ethnic minority groups (e.g. Hispanic or Latino individuals)^(21)^. The present study seeks to fill a gap in evidence. Prior studies have not (1) estimated the rates of food insecurity and overweight/obesity among NHPI adolescents using representative national data, (2) explored the association between food insecurity and overweight/obesity among NHPI adolescents using representative national data and (3) shown effects of interactions between SNAP participation and sex or food insecurity on adolescent overweight/obesity, despite findings among adults differing between women *v*. men^(16,17)^ and despite calls for further research on this topic^(18)^.

## Objectives

Using a nationally representative sample, this study’s objectives were to (1) estimate the prevalence of family food insecurity and overweight/obesity among NHPI adolescents aged 12–17 years living in the USA, (2) examine the association between family food insecurity and overweight/obesity among NHPI adolescents living in the USA and (3) explore possible factors (e.g. whether the adolescent is a girl *v*. a boy and whether the family participates in SNAP) which might have a moderation effect on the association between family food insecurity and adolescents’ overweight/obesity.

For our second objective, we hypothesised NHPI adolescents who experience family food insecurity were more likely to be overweight/obese. We hypothesised this association would differ between girls and boys, with a stronger association among girls than boys because this association has been found among adults^(16,17)^. Our third objective was exploratory; we did not posit an *a priori* hypothesis.

## Methods

### Data source and study population

This cross-sectional study on NHPI adolescents aged 12–17 years used data from the 2014 NHPI-National Health Interview Survey (NHIS)^(22,23)^. In 2014, the National Center for Health Statistics (NCHS) at the Centers for Disease Control and Prevention (CDC) launched the first NHIS focused exclusively on NHPI. Children in the sample were NHPI only or NHPI in combination with one or more other races. The questionnaire was administered to NHPI adults randomly selected from households samples from all fifty US states. A parent or guardian responded to questions about the NHPI child and the family. The surveys’ sampling procedures were designed to be representative of noninstitutionalised civilian adults and children living in the USA. The analytic dataset comprised the sample’s child-level, respondent-level and family-level data files. A detailed description of the NHPI-NHIS and NHIS can be found elsewhere^(22)^.

From February 2014 to November 2014, 479 adolescents aged 12–17 years were randomly selected. The survey’s final analytic file comprised 404 NHPI adolescents as information on seventy-five children was either not available^(24)^ or unknown^(12)^. Missing family income data reduced the present study’s analytic sample for regression analysis to 383. This study was ruled exempt as not human subject research by the [blinded for peer review] Institutional Review Board (IRB #206 591).

### Measures

Overweight/obesity is the dependent variable. The child’s weight in pounds and height in inches were reported by a parent or guardian and then converted to kilograms and meters, respectively. The weight status of children aged 12–17 years was determined using an age- and sex-specific percentile for BMI, where BMI = weight (kg)/[height (m)]^2(25,26)^. In the NHPI-NHIS, weight status was coded into four categories: underweight, normal weight, overweight and obese. Overweight/obesity was defined according to the US CDC’s BMI-for-age definition^(27)^. Overweight = BMI ≥ 85th and < 95th percentiles for children of the same age and sex. Obesity = BMI ≥ 95th percentile for children of the same age and sex.

Underweight (*n* 9) and normal weight (*n* 267) categories were recoded as non-overweight/non-obese whereas overweight (*n* 70) and obese (*n* 58) categories were recoded as overweight/obese as done in previous studies^(28)^.

Family food insecurity is the independent variable of interest. Food insecurity was assessed at the family level based on responses from an adult household member. The NHPI-NHIS measured this variable using the US Department of Agriculture’s (USDA) 10-item Adult Food Security Survey Module modified to assess food insecurity in the past 30 d^(29–31)^. Following procedures used by US National Center for Health Statistics and USDA^(30,31)^, answers of ‘Often true,’ ‘Sometimes true’ and ‘Yes’ were considered affirmative responses^(29)^. For the two items assessing the frequency of occurrence of food insecurity in the past 30 d, responses were considered affirmative if the respondent’s answer was greater than or equal to 3 d. A score (0–10) was created to represent the number of affirmative responses to the food insecurity items. For descriptive analyses, the following categories were used as follows: high food security (score = 0); marginal food security (score = 1–2); low food security (score = 3–5) and very low food security (score = 6–10)^(30,31)^. For regression analyses, food insecurity was treated as a continuous variable.

### Covariates

Additional variables included in the analysis were: age in years (continuous), sex, NHPI race (NHPI as primary race *v*. multiracial including NHPI), family structure (parents are married *v*. not), family income relative to federal poverty level (FPL) (FPL; < 100 % or ≥ 100 % of the FPL, based on poverty threshold estimates from 2013)^(30)^, educational level of the adult with the highest education in the family (≤ High school diploma/GED and > High school diploma/GED), number of children and adults in the house, family’s home ownership status (own a house or have a mortgage *v*. not) and family SNAP participation (any family member received SNAP benefits in the last calendar year *v*. not).

### Statistical analysis

The data collected in the NHPI-NHIS were obtained through a complex, multistage sample design that involves stratification and clustering. Descriptive and regression analyses were weighted based on NHPI-NHIS complex design sampling (sampling weight [wtfa_sc], stratification and primary sampling units)^(32)^. Regression analysis was based on a complete case analysis (*n* 383 due to 5·2 % missing data for the income variable). Statistical significance was determined at *α* = 0·05. STATA se 16 was used for all statistical analyses^(33)^.

### Descriptive analysis

For categorical and continuous variables, weighted percentages with corresponding 95 % CI and weighted means with corresponding 95 % CI were computed.

### Regression analysis

We used multiple logistic regression to examine the association between family food insecurity and overweight/obesity controlling for the effect of the other covariates. For the second objective and hypothesis, we included an interaction term (food insecurity × sex) in the model to capture the effect of sex. For the third objective’s exploratory analysis, we examined whether family SNAP participation would moderate the association between food insecurity and overweight/obesity by sex by adding a three-way interaction term (food insecurity × sex × SNAP) in the model.

Variance inflator indicator was computed to examine multicollinearity among independent variables. The variance inflation factor (VIF) obtained for the independent variables ranged from 1·08 to 1·51, with a mean VIF of 1·24, indicating acceptable levels of multicollinearity.

## Results

### Descriptive analysis

Table 1 presents descriptive statistics for the analytic sample (*n* 383), of which 47·1 % were girls and 52·9 % were boys. The mean age was 14·54 (95 % CI: 14·29, 14·79). Approximately a quarter (24·9 %) of the adolescents lived in families participating in SNAP, and 69·8 % of adolescents lived with parents whose highest level of education was a high school diploma or lower. The mean number of children and adults per household was 2·58 (95 % CI: 2·46, 2·71) and 2·75 (95 % CI: 2·56, 2·94), respectively.

**Table 1:** Descriptive statistics for NHPI adolescents and their Families living in the USA (*n* 383)

Measures	Mean	95 % CI	Weighted %	95 % CI
NHPI adolescent characteristics				
Age (years)	14·54	14·29, 14·79		
Girls			47·1	40·0, 54·3
NHPI (alone, not multi-race)			44·9	37·2, 52·8
Overweight/obese			33·5	25·7, 42·3
NHPI family characteristics				
Family SNAP participation (yes)			24·9	18·2, 33·0
Family income < 100 % of the 2013 FPL			15·6	11·1, 21·6
Family education (highest level)				
≤ High school diploma			69·8	61·0, 77·4
> High school diploma			30·2	22·6, 39·0
Family food security (continuous)	1·06	0·74, 1·39		
Family food security (categorical)				
High			71·4	64·1, 77·7
Marginal			12·0	8·0, 17·7
Low			8·1	5·1, 12·7
Very Low			8·5	4·7, 14·8
Family structure (married)			26·3	18·4, 36·0
Number of children in household	2·58	2·46, 2·71		
Number of adults in household	2·75	2·56, 2·94		
Family owns/Buying a house			63·2	57·4, 68·7

NHPI = Native Hawaiian or Pacific Islander. SNAP = Supplemental Nutrition Assistance Program; FPL = federal poverty level.

Data source: National Center for Health Statistics, 2014 NHPI NHIS.

A third (33·5 %) of NHPI adolescents aged 12–17 years were overweight (19·1 %) or obese (14·4 %); 8·1 % lived in families with low food security; and 8·5 % lived in families with very low food security. The mean family food security score was 1·06 (95 % CI: 0·74, 1·39), which corresponds to marginal food security.

### Regression analysis

Multiple logistic regression did not show a statistically significant association between family food insecurity and adolescent overweight/obesity. No associations were found between any other covariates and overweight/obesity, except for family SNAP participation (Table 2, Model 1). Odds of being overweight/obese were 77 % lower for adolescents in families participating in SNAP (OR: 0·23, 95 % CI: 0·08, 0·64, *P* = 0·007). The two-way interaction between food insecurity × sex was not statistically significant. However, when testing this interaction, family SNAP participation continued to be associated with reduced odds of overweight/obesity (OR: 0·22, 95 % CI: 0·08, 0·66, *P* = 0·009) (Table 2, Model 2).

**Table 2:** NHPI adolescent and family characteristics predicting adolescent overweight/obesity, results of three logistic regression models (*n* 383)

	Model 1			Model 2			Model 3		
Measures	OR	95 % CI	*P*	OR	95 % CI	*P*	OR	95 % CI	*P*
Age	1·16	0·96, 1·40	0·112	1·16	0·96, 1·40	0·110	1·18	0·97, 1·44	0·096
Girls	0·88	0·52, 1·48	0·611	0·83	0·45, 1·51	0·521	0·60	0·31, 1·17	0·130
NHPI (alone, not multi-race)	1·90	0·82, 4·38	0·129	1·88	0·80, 4·37	0·139	1·71	0·76, 3·83	0·183
Family SNAP participation (yes)	0·23	0·08, 0·64	0·007	0·22	0·08, 0·66	0·009	0·09	0·03, 0·28	< 0·001
Family education (≤ high school)	2·01	0·77, 5·20	0·145	2·02	0·78, 5·23	0·139	1·75	0·66, 4·63	0·247
Family poverty (income < 100 % FPL)	1·81	0·49, 6·75	0·361	1·87	0·47, 7·40	0·357	1·52	0·38, 6·03	0·539
Family food security (continuous)	0·96	0·82, 1·12	0·584	0·92	0·72, 1·17	0·489	0·88	0·66, 1·17	0·362
family structure (married)	0·94	0·38, 2·37	0·897	0·94	0·38, 2·36	0·894	1·05	0·42, 2·65	0·908
Family owns/buying a house	0·82	0·37, 1·84	0·624	0·82	0·37, 1·85	0·625	0·81	0·36, 1·79	0·587
Number of children in household	0·97	0·71, 1·32	0·843	0·96	0·71, 1·32	0·814	0·96	0·70, 7·32	0·794
Number of adults in household	1·07	0·81, 1·42	0·610	1·07	0·81, 1·43	0·609	1·14	0·88, 1·47	0·301
Food Security × Sex	–		–	1·07	0·78, 1·47	0·665	1·35	0·92, 1·98	0·122
Food Security × SNAP	–		–	–		–	1·36	1·00, 1·85	0·053
Sex × SNAP	–		–	–		–	9·98	2·85, 35·00	0·001
Food Security × Sex × SNAP	–		–	–		–	0·44	0·27, 0·71	0·002

NHPI = Native Hawaiian or Pacific Islander; SNAP = Supplemental Nutrition Assistance Program; Family Poverty = family income < 100 % federal poverty level (FPL), based on poverty threshold estimates from 2013. Dashes indicate variables that were not included in a model.

Data source: National Center for Health Statistics, 2014 NHPI-NHIS.

**Fig. 1:**
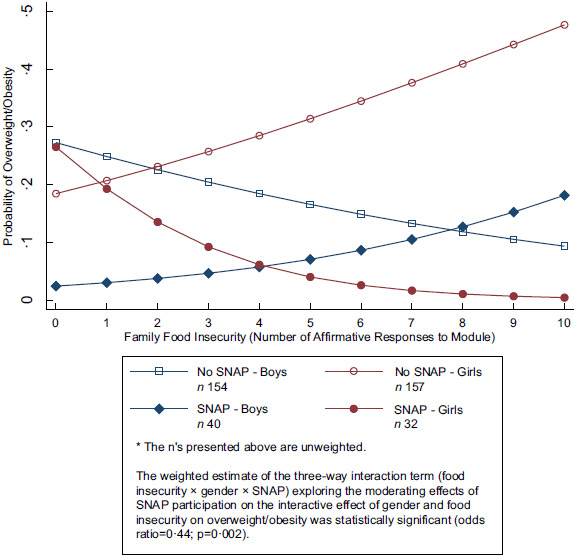
Association between Food insecurity and overweight/obesity by adolescent sex and household SNAP participation (*n* 383)

The three-way interaction (food insecurity × sex × SNAP) exploring whether family SNAP participation would moderate the interactive effect of sex and food insecurity on overweight/obesity is presented in Table 2, Model 3. The estimate of the three-way interaction term was significant (*P* = 0·002). To facilitate the interpretation of the interaction, marginal effects and plotted interactions are presented on a probability scale. For girls in SNAP families, food insecurity was negatively associated with overweight obesity. For boys in SNAP families, food insecurity was associated with overweight/obesity. For girls in families not participating in SNAP, food insecurity was associated with overweight/obesity. For boys in families not participating in SNAP, food insecurity was negatively associated with overweight/obesity (Fig. 1).

## Discussion

Among this nationally representative sample of NHPI families with adolescents, 16·6 % lived in families with low or very low food security, and 33·5 % of adolescents were overweight/obese. These findings show a higher prevalence of food insecurity than for US households with children overall (14·8 %)^(7)^ and a higher prevalence of obesity than is estimated for the general population of US adolescents of similar age range (21·2 %)^(34)^. The present study’s estimate of food insecurity (16·6 %) among NHPI families with adolescents is lower than estimates of food insecurity (20·5 %) across all NHPI households^(1)^. This finding is unexpected and not readily explained, given higher prevalence of food insecurity among US households with children than without children^(7)^.

The present study provides no evidence that family food insecurity was related to overweight/obesity among NHPI adolescents. We found no association between family food insecurity and adolescent overweight/obesity, and there was no difference between girls and boys in the relationship between family food insecurity and adolescent overweight/obesity. These null findings align with studies and systematic reviews documenting inconsistent and null associations between food insecurity and overweight/obesity among adolescents^(16–18)^. In contrast, one study showed an association between self-reported food insecurity and higher incidence of obesity among Hispanic adolescents^(21)^.

The present study provides evidence that family SNAP participation was related to lower probability of overweight/obesity for NHPI adolescents. NHPI adolescents from families participating in SNAP were less likely to be overweight/obese than those from families not participating in SNAP. This finding has not previously been documented among NHPI adolescents. Previous analysis of national^(19)^ and regional^(20)^ samples including multiple races and ethnicities have shown reductions in the probability of overweight/obesity for children and adolescents in homes participating in SNAP^(19,20)^ and shown associations between SNAP participation and better health outcomes in general^(35)^. SNAP provides increased household purchasing power for food, so this finding aligns with research showing an inverse relationship between obesity and income for adult women and for boys and girls under 18^(35)^.

Exploratory analyses showed the association between SNAP participation and lower odds of overweight/obesity was particularly pronounced for adolescent girls living within food-insecure families. To our knowledge, this interaction among food insecurity, sex and SNAP participation has not previously been documented for NHPI adolescents or for adolescents more generally. A study using data from 1979 showed SNAP participation significantly reduced the probability of being overweight or obese for adolescents boys but did not affect the probability of being overweight or obese for adolescent girls^(19)^. However, that study did not include food insecurity as a variable. Future research is needed among NHPI adolescents—and potentially among US adolescents in general—to examine whether the present study’s exploratory finding replicates. The present study’s exploratory analysis was not based on *a priori* effect size estimates that would allow for a formal power analysis and accompanying sample size justification. Interaction effects can be small and difficult to detect, and interaction effects can be challenging to replicate in future analyses. The statistical significance of the three-way interaction term in the moderation model should be interpreted through the lens of exploratory *post hoc* analysis, i.e. an additional contribution at the data analysis stage based on information provided by the current research findings but with uncertainties around the extent to which it might replicate in other samples^(36)^. If the finding replicates, future research will be needed to investigate mechanisms for why the effects of family SNAP participation on overweight/obesity might differ between adolescent boys and girls.

A primary limitation of this study is that it relies upon cross-sectional, family-member respondent-reported data for food insecurity, height, weight and SNAP participation. This limitation is shared among many survey studies, including the annual Household Food Security in the United States report^(7)^. Following a sample of households over time, documenting key variables (e.g. height, weight and SNAP participation) through more objective methods, or aligning the reference periods for the SNAP participation measure (calendar year) and food insecurity measure (past 30 d) may yield different findings than the present study. A second limitation is that the NHPI-NHIS data are from 2014. However, there is no other nationally representative survey that samples enough NHPI adolescents to characterise the key variables used in this study. A third limitation is that this study uses the term *sex* to describe the NHIS variable for which respondents identified whether an adolescent was male or female. This approach oversimplifies the complex effects of sex, gender and the interaction between sex and gender on adolescents’ BMI. It is also probable that some respondents interpreted the NHIS question about sex as being about the adolescent’s gender.

### Implications for public health

This study addresses an important gap in the literature by estimating the prevalence of food insecurity and adolescent overweight/obesity among a nationally representative sample of NHPI families with adolescents. These estimates will be of use to NHPI communities and their advocates working to address food insecurity and nutrition-related chronic disease disparities in the context of systematic data reporting gaps for NHPI health data in the USA^(37,38)^. This study contributes a novel finding that NHPI adolescents from families participating in SNAP were less likely to be classified as overweight/obese compared with those from families not participating in SNAP. Research is needed to determine the extent to which SNAP-eligible NHPI families are enrolling in SNAP and whether interventions to increase enrollment would improve NHPI adolescents’ health outcomes.
